# Evaluation of Low-Molecular-Weight Heparin Treatment on First- and Second-Trimester Screening Test Results

**DOI:** 10.7759/cureus.35137

**Published:** 2023-02-18

**Authors:** Mehmet AK, Bertan Demir, Cevat Rifat Cundubey, Seyma Cam

**Affiliations:** 1 Obstetrics and Gynecology, Kayseri City Training and Research Hospital, Kayseri, TUR

**Keywords:** trisomy, down sendromu screening test, pregnancy, low-molecular weight heparin (lmwh), thrombophilia

## Abstract

Background

The serum markers used in first- and second-trimester screening tests can be affected by different causes such as smoking, infertility treatment, and the presence of diabetes mellitus, which should be considered by obstetricians when giving information to patients. Low molecular weight heparin (LMWH) has a critical importance in the prophylaxis of deep vein thrombosis both in the antenatal and postnatal period. The aim of the current study is to investigate the effect of LMWH use on the first- and second-trimester screening results.

Methods

A retrospective analysis in our outpatient clinic between July 2018 and January 2021 of first- and second-trimester screening test results was conducted to assess the impact of LMWH treatment for patients with thrombophilia who started LMWH after pregnancy was detected were included. Test results were obtained as a multiple of median (MoM) combined with ultrasound measurements, maternal serum markers, and maternal age in addition to the nuchal translucency first-trimester test.

Results

The pregnancy-associated plasma protein-A (PAPP-A) MoM was lower and alpha-fetoprotein (AFP) and unconjugated estriol (uE3) MoMs were higher in patients treated with LMWH than in the control group (0.78 MoM vs 0.96 MoM; 1.00 MoM vs 0.97 MoM; and 0.89 MoM vs 0.76 MoM, respectively). Human chorionic gonadotropin (HCG) levels did not differ between groups at either time point.

Conclusions

Treatment of pregnant women with LMWH for thrombophilia may change the MoM values of serum markers for both first- and second-trimester screening tests. Obstetricians should be aware of this when advising screening tests to thrombophilia patients and should consider offering fetal DNA tests for this group instead.

## Introduction

Thrombophilia is a condition that predisposes a person to thrombosis, which can occur with inherited or acquired diseases as well as contribute to adverse outcomes in pregnant women. During pregnancy, hypercoagulation occurs to prevent postpartum bleeding. Although not all thrombophilia patients experience thrombotic complications during pregnancy, half of the patients with thromboembolism during pregnancy have thrombophilia [1]. The most observed causes of congenital thrombophilia are the Factor V Leiden (FVL) mutation, Prothrombin G20210A (also called prothrombin gene mutation [PGM]) gene mutation, Protein C (PC) deficiency, Protein S (PS) deficiency, and Antithrombin (AT) deficiency FVL and PGM together account for 50% to 60% of the hereditary cases (primary) with a hypercoagulative state in Caucasian populations [2]. Deficiencies in PS, PC, and AT make up the majority of the remaining Although prevalent collectively in about 10% of the Caucasian population, these disorders appear to be partially responsible for almost half of the maternal venous thromboembolisms (VTEs) [3]. Individuals with the following hereditary thrombophilia are at higher risk for VTE:AT deficiency, FVL homozygotes, PGM homozygotes, compound heterozygous FVL, and heterozygous PGM [4].

During pregnancy, antiphospholipid syndrome (APS) is the most common cause of acquired thrombophilia. It is diagnosed through the detection of anti-phospholipid antibodies including lupus anti-coagulant (LAC) antibodies, anti-cardiolipin antibodies (aCL), or anti-β2-glycoprotein antibodies (aβ2GPI) [5]. APS is associated with fetal death after 10 weeks of gestation, recurrent embryonic loss, and 10%-15% of women experiencing a fetal death beyond 20 weeks of gestation [6]. Most adverse pregnancy outcomes, such as miscarriage, fetal demise, fetal growth restriction, placenta abruption, and preeclampsia, are at an increased risk in mothers with inherited thrombophilia [7].

Pregnancies at risk of trisomy 21, 18, and 13 can be screened and identified early using the first-trimester combined test and second-trimester triple test [8]. In the first trimester combined test, a sonogram is used for NT determination, and the biochemical markers of aneuploidy (pregnancy-associated plasma protein A (PAPP-A) and free-beta or total hCG are measured at 11+0 to 13+6 weeks of gestation. In the second trimester, the triple test measures the maternal serum markers alpha-fetoprotein (AFP), free or total hCG, and unconjugated estriol (uE3) from 15+0 to 22+6 weeks of gestation in combination with maternal age [9]. The laboratory's specific cut-off value (e.g., ≥ 1/250) determines any increased risk of fetal Down syndrome. These markers are associated with different adverse pregnancy outcomes [8,9]. Although treated with low molecular weight heparin (LMWH), studies show that APS thrombophilia causes changes in the triple test values [10]. Some studies suggest that multiple medians (MoM) levels of PAPP-A, free b-hCG, and NT are altered in women with inherited thrombophilia [11]. Studies have shown that LMWH used in patients with thrombophilia not only has an anticoagulant effect but also has anti-inflammatory and immunomodulatory effects. LMWH positively affects trophoblast migration and invasion through different mechanisms and molecules. In addition, there are publications that show increased LMWH drives trophoblast proliferation and therefore, hCG release. Although it is not observed with the use of high molecular weight heparin, we aim to investigate the effect of heparin use on the first- and second-trimester screening results.

## Materials and methods

In this study, patients with inherited or acquired thrombophilia that used prophylactic LMWH prior to 12 weeks gestation between July 2018 and January 2021 were evaluated retrospectively at the prenatal outpatient unit of a tertiary referral hospital in Kayseri, Turkey. The study group was composed of singleton pregnancy cases that suffered fetal loss later than 12 weeks, had more than three spontaneous abortions, had a history of preeclampsia leading to premature birth or fetal growth restriction, and were treated with LMWH. LMWH doses are adjusted in relation to weight of pregnant women. Patients with results from the first-trimester combined test and second-trimester triple test were included. Women with singleton pregnancies who did not have a positive test for inherited or acquired thrombophilia or have a previous thrombophilia history (recurrent abortions, deep vein thrombosis, pulmonary embolus, etc.) were included in the control group. A total of 16,900 pregnant women with singleton fetuses were screened in the first and second trimesters for combined aneuploidy at our clinic. After scanning data to evaluate first-trimester screening tests, a total of 263 women had inherited or acquired thrombophilia and were considered the study group, whereas, 342 pregnant women were selected for the control group. The evaluation of the second-trimester screening test found an additional 140 had thrombophilia, who was assigned to the second study group, and an additional 282 women were included in the second control group. The diagnosis of hereditary thrombophilia was made according to identifying a FVL mutation, Prothrombin G20210A mutation, PS deficiency, PC deficiency, or AT deficiency. The diagnosis of APS, the most commonly acquired thrombophilia, was made by the detection of anti-cardiolipin antibodies (aCL) (IgG and IgM by enzyme-linked immunosorbent assay [ELISA]), anti-beta2-glycoprotein (GP) I antibodies (IgG and IgM by ELISA), and lupus anti-coagulant (LA testing). All patients' LMWH usage dose, first trimester combined with triple test serum marker results, reasons for using LMWH, and the first-trimester nuchal translucency (NT) and Crown-rump length (CRL) measurements were recorded. The study was approved by the ethics committee for research at Kayseri City Hospital with approval number 8.1.2021/46. The first trimester (performed at 11+0 to 13+6 weeks of gestation) combined test included NT sonographic measurements and assessment of the biochemical markers PAPP-A and free-beta or total hCG. The triple test, used to estimate risk in the second trimester (performed at 15+0 to 19+6 weeks of gestation), was performed using the typical maternal serum markers AFP, free or total hCG, and uE3 in combination with maternal age. These markers were reported as MoMs. The patient's weight, history of diabetes, smoking, and in vitro fertilization records were considered when calculating the MoM value. Patients with a chromosomal anomaly risk higher than 1/300 were offered chorionic villus sampling (CVS) or amniocentesis (A/S). Any patients receiving a fetal aneuploidy diagnosis were excluded from the study.

The conformity of the data to a normal distribution was evaluated with histograms, q-q graphs, and the Shapiro-Wilk test. The homogeneity of variance was tested with Levene's test. Welch’s two-sample t-test was applied to account for the homogeneity of variance for normally distributed data with quantitative variables in comparisons between paired groups. The Mann-Whitney U test was used for the variables with a non-normal distribution. Normally distributed data were expressed as mean ± standard deviation and non-normally distributed data were expressed as median (1st-3rd quarter). The analysis of the data was carried out in TURCOSA (Turcosa Analytics Ltd Co, Turkey, www.turcosa.com.tr) statistical software. A p-value of less than p<0.05 was considered statistically significant.

## Results

Comparisons were made between the first trimester patient group (n:263) and the control group (n:342) for age, weight, PAPP-A MoM, b-hCG level, and CRL (Table 1). No statistically significant difference was determined between the patient and control groups in b-hCG level (p>0.05). Mean age, body weight, and CRL in the thrombophilia group were 29.1 years, 73.94 kg, and 88 mm in the study group and 27.4 years, 66.2 kg, and 64 mm in the control group. The age, weight, and CRL values were significantly higher in the patient group than in the control group (p<0.05). The PAPP-A MoM value was significantly lower in the patient group than in the control group (0.78 vs 0.96 MoM, respectively, p<0.05).

**Table 1:** Comparisons of the first-trimester paired test results of the patient and control groups Human chorionic gonadotropin = hCG, Pregnancy-associated plasma protein-A = PAPP-A, multiple of median = MoM, crown-rump length = CRL

Variable	Group		p-value
	Patient (n=263)	Control (n=342)	
Age (years)	29.18±5.96	27.46±5.77	<0.001
Weight (kg)	73.94±16.81	66.23±12.97	<0.001
PAPP-A MoM	0.78(0.53-1.15)	0.96(0.64-1.32)	0.001
b-hCG MoM	0.86(0.65-1.22)	0.86(0.61-1.29)	0.893
CRL (mm)	88.0(85.0-91.0)	64.0(58.15-69.65)	<0.001

Comparisons were made between the second-trimester patient group (n:140) and the control group (n:282) for age, gestational day, weight, AFP, E3, and b-hCG levels (Table 2). No statistically significant difference was determined between the patient and control groups in age, gestational day, and b-hCG level (p>0.05). The weight, AFP, and E3 values were significantly higher in the patient group than in the control group (75.1 kg vs 65.6 kg, 1.0 vs 0.89 MoM, and 0.97 vs 0.76 MoM, respectively, p<0.05).

**Table 2:** Comparisons of the second-trimester three-way test results of the patient and control groups Human chorionic gonadotropin = hCG, unconjugated estriol = uE3, alpha-fetoprotein = AFP, multiple of median = MoM

Variable	Group		p
	Patient (n=140)	Control (n=282)	
Age (years)	28.29±6.08	27.20±5.70	0.073
Gestational day	119.03±7.30	119.98±7.35	0.209
Weight (kg)	75.15±16.47	65.64±14.01	<0.001
AFP MoM	1.00(0.78-1.32)	0.89(0.69-1.09)	<0.001
uE3 MoM	0.97(0.79-1.19)	0.76(0.61-0.95)	<0.001
b-hCG MoM	0.84(0.59-1.25)	0.86(0.64-1.11)	0.915

## Discussion

Screening tests for Down Syndrome have been widely used in the first and second trimesters for many years. Parameters used in the first-trimester screening test are PAPP-A, b-hCG, and fetal NT [12]. In the second-trimester screening test, AFP, b-hCG, uE3, and fetal biparietal diameter have been used [13]. In addition, maternal age is used in risk calculation [14,15]. These tests have low sensitivity and specificity and are affected by conditions such as IVF pregnancy, smoking, obesity, and diabetes. In this study, we aimed to investigate whether LMWH treatment used in pregnant women with thrombophilia is among the parameters affecting these screening tests.

PAPP-A, which is used in the first-trimester test, is secreted from placental syncytiotrophoblast cells and decidua and decreases in trisomy 21, while b-hCG is high [16,17]. b-hCG also is elevated in trisomy 13 and 18. AFP is high in fetal blood and increases in neural tube defects but decreases in Down syndrome. uE3 is released from the fetal placenta and decreases in Down Syndrome. PAPP-A can also predict adverse neonatal outcomes such as intrauterine growth restriction and preeclampsia [18,19]. In studies performed in recurrent spontaneous abortions, PAPP-A levels were lower than in normal pregnant women. In the first-trimester screening test part of our study, PAPP-A was significantly lower in the patient group (p<0.001). We concluded that LMWH treatment did not have a positive association with PAPP-A levels.

High AFP levels have also been associated with thrombophilia [20]. AFP elevation is also associated with placental anomalies. In the second trimester comparison, AFP measurement levels were significantly higher in the patient group. Although one previous study showed no differences, our study had results similar to Brochet et al.

uE3 originates from 17 OH-DHEAS, is secreted from the fetal adrenal gland, and is affected by placental sulfatase activity [21]. Although uE3 levels were low in other studies, the high value in our study indicates the regulatory role of LMWH in the treatment. Patients with hereditary thrombophilia had lower uE3 MoM values ​​in triple test parameters, and as a result, the risk of trisomy 21, trisomy 18, and trisomy 13 may be overestimated and patients may be exposed to unnecessary invasive diagnostic procedures [22]. Second-trimester maternal serum screening may indicate higher hCG serum levels in women with acquired thrombophilia, even those treated with LMWH in early pregnancy, suggesting the possibility of placental dysfunction rather than fetal aneuploidy [23].

Studies have shown that LMWH used in patients with thrombophilia not only has an anticoagulant effect but also has anti-inflammatory and immunomodulatory effects. LMWH positively affects trophoblast migration and invasion through different mechanisms and molecules. In addition, there are publications that show increased LMWH drives trophoblast proliferation and therefore, hCG release, although it is not observed with the use of high molecular weight heparin. In our study, no difference was found between the patient group and the control group's hCG levels. Whether this effect is dose-dependent or not is an issue that needs to be investigated.

The limitations of this study are that the test results may be affected by the subtypes of hereditary thrombophilia. In addition, it is not clear whether the effect observed in the test results is due to the use of LMWH or thrombophilia. However, discontinuation of LMWH would not be ethical in patients with difficulty to differentiate thrombophilias.

## Conclusions

In summary, we found lower PAPP-A and higher AFP and uE3 in women that used LMWH. Although some of the findings in our study were similar to other publications, some results were different. This may pose difficulties for evaluation, especially in the population at high risk for Down syndrome. More work is needed to confirm these results. Because of these unknowns, clinicians should be careful in the follow-up of patients with thrombophilia and have an open dialogue with a perinatologist if necessary. Treatment of pregnant women with LMWH for thrombophilia may change the MoM values of serum markers for both first- and second-trimester screening tests for Down Syndrome. Obstetricians should be aware of this when advising screening tests to thrombophilia patients and should consider offering fetal DNA tests for this group instead.
